# HIV-1 Persistence in Children during Suppressive ART

**DOI:** 10.3390/v13061134

**Published:** 2021-06-12

**Authors:** Mary Grace Katusiime, Gert U. Van Zyl, Mark F. Cotton, Mary F. Kearney

**Affiliations:** 1 HIV Dynamics and Replication Program, CCR, National Cancer Institute, Frederick, MD 21702, USA; kearneym@mail.nih.gov; 2 Division of Medical Virology, Stellenbosch University and National Health Laboratory Service Tygerberg, Cape Town 8000, South Africa; guvz@sun.ac.za; 3 Department of Pediatrics and Child Health, Tygerberg Children’s Hospital and Family Center for Research with Ubuntu, Stellenbosch University, Cape Town 7505, South Africa; MCOT@sun.ac.za

**Keywords:** HIV-1 persistence, perinatal HIV-1, HIV-1 reservoirs, children, long-term ART

## Abstract

There is a growing number of perinatally HIV-1-infected children worldwide who must maintain life-long ART. In early life, HIV-1 infection is established in an immunologically inexperienced environment in which maternal ART and immune dynamics during pregnancy play a role in reservoir establishment. Children that initiated early antiretroviral therapy (ART) and maintained long-term suppression of viremia have smaller and less diverse HIV reservoirs than adults, although their proviral landscape during ART is reported to be similar to that of adults. The ability of these early infected cells to persist long-term through clonal expansion poses a major barrier to finding a cure. Furthermore, the effects of life-long HIV persistence and ART are yet to be understood, but growing evidence suggests that these individuals are at an increased risk for developing non-AIDS-related comorbidities, which underscores the need for an HIV cure.

## 1. Introduction

A total of 1.8 million children aged 0–14 years were living with HIV as of 2019 [1]. Of these, only 53% had access to antiretroviral therapy (ART). Furthermore, 15% of pregnant women are not on ART to prevent transmission to their unborn child. Vertical HIV transmission can occur in utero, intrapartum, or postnatally via breastfeeding. In rare cases, horizontal transmission also occurs through surrogate breastfeeding, premastication of food, and healthcare-associated transmissions [2,3,4,5]. Despite this, maternal viral load suppression by ART has been instrumental in the 52% decline in vertical transmission since 2010 [1]. Nonetheless, there are still 150,000 new HIV infections in children aged 0-14 years every year, far above the WHO target of zero new infections among infants by 2020 [1,6]. Meeting these targets may be even more challenging given the impact of the COVID-19 pandemic on access to HIV testing and ART, particularly in the developing world where 90% of vertical transmission occurs [7].

All 1.8 million children infected with HIV worldwide must maintain life-long ART, as there is no cure for HIV. Adherence to ART is a growing challenge in pediatric HIV care and widely influenced by a host of socio-economic and psychological factors [8,9]. Furthermore, cumulative drug toxicities and non-AIDS-related morbidities in those living with HIV and treated since birth are largely unknown. In older people living with HIV, an increased risk of developing ART-related morbidities such as diabetes, hypertension, and cardiovascular disease, among others, has been observed [10,11,12,13,14]. These ART-related morbidities may occur in individuals born with HIV as they progress in age, along with other unknown complications related to life-long infection and treatment. 

The changes to the WHO treatment guidelines to accommodate all children living with HIV regardless of CD4 threshold, coupled with the increase in life expectancy from effective ART, resulted in national healthcare budgets having to cater to lifelong ART [15]. The high cost of treating over 1.8 million individuals (and counting) is not economically sustainable, particularly in the developing world where most of the disease burden exists. For these reasons, a cure is warranted. 

During HIV-1 replication, viral RNA is reverse-transcribed to cDNA before integration into the host cell genome. In most cells, HIV infection leads to active replication, resulting in the release of new virions and the destruction of the infected cell. However, in a subset, replication-competent proviruses can remain dormant within the host cell’s genome, enabling HIV to persist in this form for the lifespan of the infected cell [16,17]. These cells remain hidden from the effects of the immune response, are unaffected by antiretroviral drugs (which target various stages of viral replication), and can reactivate to produce new cycles of replication when therapy is interrupted. Such cells are referred to as latent reservoirs and are the major barrier to cure in ART-suppressed individuals [18]. This review aims to provide an overview of HIV-1 reservoir establishment, persistence, and long-term effects in ART-suppressed children.

## 2. Reservoir Establishment in Early Life

In the absence of ART, 50% of children born with HIV die by the age of two years. In contrast, people who acquire HIV as adults typically survive three or more years without treatment. This difference provides evidence that the environment in which HIV infection is established in children and adults is clinically relevant [19,20]. In children, HIV is introduced into a largely immunologically inexperienced environment [21]. The immune system in early life supports decreased cytokine production, leading to a hypo-inflammatory innate immune response [22]. Additionally, infant antibody responses are generally delayed at the onset, have a lower peak, persist for shorter periods, and have less affinity maturation and heterogeneity [23,24]. Furthermore, the majority of CD4+ T cells in infants are naïve and quiescent with CD4+CCR5+ expression on cell surfaces, not reaching adult levels until after 5 years of age [25,26]. The absolute number and percentage of CD4+ T cells in healthy children is higher than in adults and gradually declines to reach adult levels by 6 years of age (median of 54% at birth declines to 37% by 6–12 years) [27,28]. Thymopoiesis is more active in early life, and thymic involution begins after puberty. In long-term-treated children living with HIV, thymic volume and activity appears to be retained and comparable to peers without HIV [29,30]. As a result, children on ART appear to have better immune reconstitution than infected adults due to the role of thymic output in repopulating naïve cells [31].

Little is known about the exact cell subsets infected by HIV during early life. Current assays that quantify HIV reservoirs measure one or more of the following: (i) infected cells—the total number of cells harboring HIV-1 DNA, (ii) inducible reservoirs—cells that produce detectable viral nucleic acid or particles on stimulation (but may not replicate), and (iii) replication-competent reservoirs—cells that produce viruses upon stimulation and then complete successive replication cycles ex vivo [32]. In adults, in vivo studies with a few participants on long-term ART have shown that although HIV primarily infects long-lived memory T cell subsets (i.e., central memory and stem memory), naïve cells may be an equal or greater contributor to the inducible, replication-competent reservoir [33,34,35,36]. In children, although the majority of T cells are naïve and early ART results in a limited number of detectable infected cells, the transitional memory compartment appeared to harbor a larger proportion of HIV-1 DNA than the central memory T cell compartment in a small study cohort [37]. The relative distribution of infected, inducible, and replication-competent HIV in various cell subsets remains to be shown in early-treated children, probably due to the small blood volumes and the rapid decay of infected cells in the population [38].

**Figure 1:**
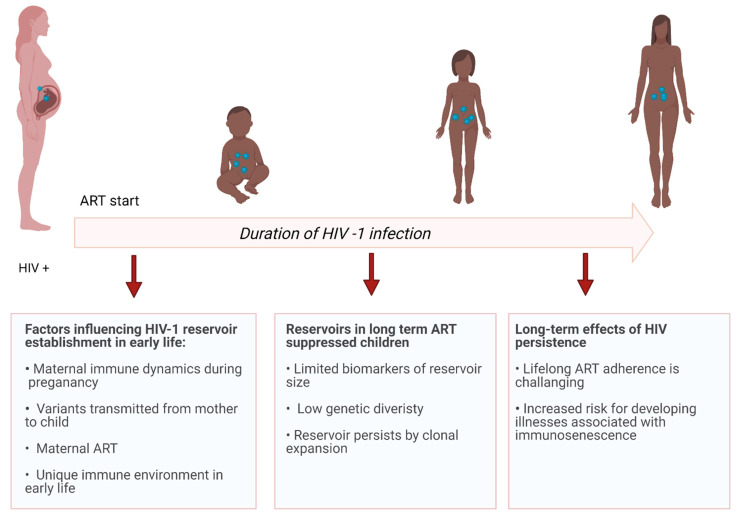
HIV-1 persistence in perinatally infected individuals.

Studies have shown an association between viral load and biomarkers of reservoir size, indicating that reservoir seeding occurs during uncontrolled viral replication [39,40]. In the pre-ART era, there was an association between maternal viral load at delivery and neonatal pre-ART viral load, where infants had viral loads as high as 10^6^ HIV RNA copies/mL of plasma—comparable to levels in untreated adults [41,42,43]. With widespread access to ART, treatment that crosses the placenta may contribute to lowering pre-ART viral loads in children who become infected, to a median of 10^4^ HIV RNA copies/mL of plasma, potentially limiting the establishment of long-lived reservoirs [41,42,43]. Similar to adults, vertical transmissions occur over a selective genetic bottleneck, with infection most often established by a single variant [44,45,46]. Recent studies comparing HIV V3 envelope sequences from mother–infant transmitting pairs found that transmitted founder variants in infants are resistant to neutralization by maternal broadly neutralizing antibodies (bNabs) compared with non-transmitted variants in the mother, indicating that maternal bNabs can select for resistant variants that establish infection in the child [47,48,49]. Maternal immune dynamics during pregnancy, the variant(s) transmitted, maternal ART, the timing of infant ART initiation, and the unique immune environment in early life all contribute to the reservoir that is established in children with perinatally acquired HIV (Figure 1) [50].

## 3. Reservoirs in Early-Treated, Long-Term Suppressed Children

The high mortality reported among children who are born with HIV and initiate ART only when symptomatic suggests that earlier therapy is required to prevent disease progression [19,51]. To this end, in the Children with HIV Early antiRetroviral therapy (CHER) randomized trial conducted in South Africa determined that initiating ART within 12 weeks of birth, when the immune system was most immature, resulted in long-term benefits including slower disease progression and limited reservoir size [52,53].

### 3.1. HIV-1 Cell-Associated DNA: A Biomarker of the Reservoir in Long-Term-Treated Children

Quantitative PCR (qPCR) measurements of reservoir biomarkers, particularly total HIV-1 DNA (proviral load), are commonly used to estimate levels of HIV persistence during ART suppression [54,55,56,57]. Cohort studies of adults showed that early ART limits reservoir seeding particularly in long-lived CD4+ T cell subsets [58]. Likewise, several cross-sectional studies have compared proviral loads during long-term ART in early-treated vs. later-treated children. Ananworanich et al. found that after 6 years on ART, children who initiated within 6 months of birth had low proviral loads (median total HIV DNA 132 copies/10^6^ CD4+ T cells; range 11-1804) [50]. A similar study found that children who initiated ART within 3 months of age had six-fold lower proviral loads than those who initiated after 3 months but within a year of life [59]. Luzuriaga et al. compared children who initiated ART within the first year of life with those who initiated after 4 years and found significantly higher proviral loads, recoverable replication competent virus, T cell activation, and slower proviral decay in the latter [37]. A study of 20 children from the CHER cohort who had viremia well-suppressed on ART for 7–8 years also found significantly lower DNA and RNA loads in those who initiated treatment within two months of birth compared with those who initiated later [53]. These findings are mirrored by others [60,61,62,63,64,65,66,67] and highlight the role of early ART in limiting the size of the HIV reservoir. This finding is well-illustrated by a recent study in children showing a positive correlation between HIV RNA area under the viral load curve in the first year of life and HIV DNA levels at one year of age [68]. Two other studies in children reported that time to first viral load suppression was strongly and positively associated with infectious provirus levels over the first two years of life [62,69].

### 3.2. Measuring Infectious Virus in Long-Term Treated Children

qPCR approaches have the benefit of being highly sensitive, high-throughput, relatively inexpensive, and requiring low sample volumes. However, the majority of proviral HIV is defective and total HIV-1 DNA assays do not distinguish between genetically defective or intact proviruses due to the high background level of defective proviruses when on ART [70]. HIV DNA qPCR assays overestimate the true size of the reservoir by more than 300-fold [70] and have high variance and bias due to the different genes and gene regions being targeted [32]. The quantitative viral outgrowth assay (qVOA) was once the gold standard for measuring the replication-competent HIV reservoir [71,72] but is now being replaced or used in conjunction with the Intact Proviral Detection Assay (IPDA) (more details on IPDA are provided below) [73]. qVOA involves the mitogenic reactivation of resting CD4+ T cells by limiting dilution in the presence of feeder cells to enable subsequent rounds of infection [32,71,74]. Maximum likelihood statistics are then used to estimate the amount of reactivated virus as infectious units per million (IUPM) [72]. There are some limitations with qVOA: it is expensive, time consuming, and requires large blood volumes, and is thus not ideal for use in pediatric cohorts. Furthermore, qVOA underestimates the reservoir size by 25-fold in adults treated during acute infection and 27-fold in those treated during chronic infection because not all genetically intact proviruses are sufficiently reactivated in vitro and may require multiple rounds of stimulation [75,76]. In a study of 14 children treated at the median age of 2 months, infectious virus was detectable by qVOA in 60% of the children after 1.8 years on ART [69]. However, when children treated around the same age were tested for infectious virus at 11.1 years on ART, none was detected, showing that HIV-1 reservoirs in early-treated children declined to levels that were undetectable by qVOA during long-term ART [77]. Further illustrating this same finding is a more recent study that compared the inducibility of viral reservoirs between perinatal and adult infection and found fewer replication-competent proviruses persisting in children on long-term ART [78]. Because the blood draws in the children were smaller, resulting in fewer cells available for these assays than in adults, comparisons between the two groups were normalized for cellular input.

### 3.3. Post-ART Control of Viral Replication

The case of the “Mississippi baby”, who initiated ART within 30 h of life and showed no evidence of HIV replication for a period of 27 months without treatment, indicates a very low reservoir and supports the idea that early-treated infants may be ideal candidates for curative interventions [79,80,81]. Other recent cases of children who, following very early ART, had delayed viral rebound with no detectable virus in peripheral blood despite no evidence of immune-mediated control further highlight the role of early ART in limiting reservoir establishment [80,81,82]. There is evidence to suggest that, in some children, early ART can lead to post-treatment control of viral replication. For instance, post-treatment control was reported for a South African child from the CHER cohort who was diagnosed with HIV at 32 days of age and initiated time-limited ART at 8.5 weeks of age for the next 40 weeks [83]. ART was then interrupted, as per the trial, and the child remained aviremic for 9 years [83]. Another French child maintained treatment control for more than 11 years after treatment interruption. The child was diagnosed at 4 weeks of age and started ART at 3 months of age until between 5.8 and 6.8 years of age when the family discontinued treatment [82]. The immune-mediated mechanisms by which post-treatment viral control is achieved in such cases are yet to be understood. Whereas adults who started ART during Fiebig stage I had rapid viral rebound [84], up to 15% of adults from the VISCONTI cohort and the SPARTAC study, who initiated ART at a median of 10 weeks after diagnosis of primary HIV infection in VISCONTI, and 12 weeks after seroconversion in SPARTAC, were able to maintain viral load control after treatment cessation [82,85]. This suggests that whereas early infection limits the reservoir size and prevents immune exhaustion, a period of viral replication is required for an immune response to develop, which may contribute to post-treatment control [86].

### 3.4. Genetic Diversity during Long-Term ART

The genetic diversity of viral populations within infected children and adults is initially low (mean intra-patient genetic variation of 0.96% in HIV-1 gag gene) due to the genetic bottleneck that establishes infection by one or a few variants [37,80,87,88,89,90,91,92]. However, these populations quickly diversify because of the rapid uncontrolled replication involving error-prone reverse transcriptase and viral escape from immune pressure [91]. To assess the effects of early ART on genetic evolution in the reservoir, a longitudinal study of 12 children who started ART within 2 months of age found limited evolution in env, gag, and pol sequences after about 5.5 years on suppressive ART [90]. A similar study from the CHER cohort, comparing gag-pol sequences from pre-ART to 7–8 years on ART, utilized three phylogenetic and statistical tests to assess changes in viral population diversity and divergence between pre-ART and 7–8 years on ART. There was no evidence of viral evolution in eight fully suppressed children, whereas in the two partially suppressed children, all three tests showed significant evidence of viral evolution [61]. Moreover, the same study found a significant positive association between HIV-1 proviral loads after 7–8 years on ART and proviral diversity (calculated as average pairwise distance), suggesting that the higher the proviral load, the more diverse the viral population [61]. Similar findings were observed in adults where comparisons of sub-genomic sequences from plasma before, during, and after ART interruption showed no significant difference between proviruses prior to ART and rebounding plasma virus after ART interruption, suggesting that ART halts the cycle of ongoing replication that may lead to genetic diversification [93,94].

### 3.5. Reservoir Decay Kinetics and the Proviral Landscape during Long-Term ART

The decay of proviral DNA after initiation of ART shapes the proviral landscape in long-term ART. In children who initiate ART early, HIV-1 DNA decays rapidly, likely due to the death of productively infected cells, loss of unintegrated virus, and concurrent blocking of newly infected cells [37,95]. Veldsman et al. recently showed that HIV-1 DNA levels (measured by qPCR) in 3/11 children who initiated ART within 8 days of birth reached undetectable levels between 6 days to 3 months on ART, while levels in the others continued to decline for a mean half-life of 64.8 days over the first 12 months [38]. Other studies in children who initiated ART between 0.6 and 3 months of age report an HIV DNA half-life of 53–107 days [68,96]. Similar to children, longitudinal qPCR measurements of proviral DNA in adults after ART initiation reveal that the steepest decay in HIV DNA occurs in the first year of ART [58,97,98,99,100]. However, longitudinal measurements of the inducible reservoir taken by qVOA reveal a slower half-life of 3.6 years [101,102]. More recently, the novel IPDA assay was used to study proviral decay in adults [73,103,104]. IPDA uses droplet digital PCR to target two conserved regions in the viral genome, enabling the simultaneous quantification and discernment of intact and defective genomes [73]. These studies reported differential decay between intact and defective genomes, where intact genomes appeared to decay more rapidly in the first 7 years on ART with a half-life of 4.0 years, similar to decay measurements taken by qVOA [73,102,103,104]. In children treated within 2 weeks of birth, IPDA measurements recorded at 2 years on ART suggested that intact proviruses decayed significantly faster than defective genomes during the first 24 weeks on ART [105].

Although it has long been known that most proviral HIV DNA that persists on long-term ART is defective, a recent study in adults utilizing near full-length proviral sequencing showed that these defects accumulate within weeks of infection [75]. In adults initiating ART during chronic infection, 98% of proviral DNA was defective while 92% was defective in those treated during the acute infection phase [75]. Of these defective genomes, 9–50% contained guanine-to-adenine (G to A) hypermutations caused by cytidine deaminases APOBEC3G and APOBEC3F, which act as HIV restriction factors [70,73,106]. Large internal deletions account for 45–68% of defects due to template switching during reverse transcription. Other defects include frameshifts and nonsense mutations introduced during reverse transcription [70,73,106,107,108,109]. However, a larger follow-up study involving 400 diverse adult participants suggested that intact proviruses by IPDA are four-fold higher than previously reported [110]. In early-treated, long-term-suppressed children, proviral structures were reported to be similar to adults assayed by near-full-length proviral sequencing. The largest proportion again has internal deletions and/or hypermutation and only up to 1% are genetically intact [77,111].

## 4. Clonal Expansion Maintains the Reservoir in Treated Children

In early-treated, long-term, virally suppressed children, identical sub-genomic HIV proviral sequences were detected in PBMCs and sputum and increased in proportion over time [87]. Similarly, in adults, single-cell genome sequencing revealed identical viral sequences in resting CD4+ T cells that persist in various anatomical sites and rebound after treatment interruption [94,112,113,114,115]. An assay targeting the 3′ end of HIV and the adjacent human genome found that these identical HIV sequences also had identical sites of integration in the host, thus demonstrating the proliferation of HIV-infected cells [116]. Advances in technology have since enabled targeted, high-throughput amplification and sequencing of proviral sites of integration and revealed that latently infected cells can have identical integration sites [116,117,118,119,120]. Furthermore, even though most proviral DNA is defective, expanded cell clones can harbor intact proviruses that produce infectious virions and lead to non-suppressible viremia in ART adherent individuals [76,118,121,122,123]. In fact, during suppressive ART, at least 40–80% of infected cells are in expanded clones, implicating clonal expansion as the major mechanism maintaining the reservoir during ART [118,120,124,125,126].

In perinatally infected children in the CHER cohort, HIV-infected cell clones were detectable before ART initiation and remained detectable up to 9 years on suppressive treatment. In another study, a child who initiated treatment within the first week of life had a potential clone detected 7 hours after birth, suggesting that early ART alone does not interfere with the proliferation of infected cells [105,127]. In adults, clones expand to detectable levels by 4 weeks post-infection (Fiebig stage V) [128]. Based on these data, mathematical models predict that 75–95% of cells comprising the HIV reservoir are the result of cellular proliferation [128,129].

T cell proliferation can be driven by homeostatic stimuli, such as IL-5 and IL-7, that induce cell division without reactivation of resting cells [36,76]. Rarely, the site of proviral integration in the host genome, particularly sites in genes responsible for cell growth and survival, may drive the proliferation of infected cell clones or, at least, promote their survival [116,120,124,130,131]. In children, selection for integrations in STAT5B and BACH2 was observed both prior to and during long-term ART [127]. However, antigenic stimulus is likely the greatest contributor to the persistence of infected clones. In adults, infected cell clones wax and wane in response to both homeostatic and antigenic stimuli [118,132]. T cell receptor β chain (TCRβ) sequencing paired with integration site analysis reveals that proviral populations in cells responding to chronic viral antigens like CMV, EBV, and influenza are dominated by clones that expand to levels higher than expected by homeostatic proliferation [133,134]. These findings are of particular concern in pediatric cohorts from geographical regions where the antigenic burden is high. In Gambia, two-thirds of infants have CMV infection by 3 months of age and 85% by 12 months of age [135]. In Zimbabwe, a study involving 400 children and adolescents living with HIV also found clinically significant levels of CMV DNAemia (>1000cp/mL) in 14.7% of participants [136]. These findings suggest that clonal selection driven by antigenic stimuli may be a major driver of HIV persistence in children from the developing world.

## 5. Long-Term Effects of HIV Persistence in Perinatally Infected Individuals

Although modern ART has revolutionized disease outcomes for children with perinatally acquired HIV, there is growing concern about the consequences of life-long HIV persistence for these individuals. In treated adults, chronic inflammation and immune activation leads to non-AIDS defining comorbidities including cardiovascular, neurocognitive, and metabolic disorders [137]. Due to a longer duration of the persisting infection, adolescents and young adults who acquired HIV in childhood are at an even greater risk for developing illnesses associated with immunosenescence. ART reduces immune activation in children, but levels remain higher than in their uninfected counterparts [138]. Childhood vaccinations and exposure to coinfections may contribute to chronic immune activation [135,136,139,140,141].

A recent study comparing the frequency of immune cell subsets in young adults with perinatally acquired HIV (aged 18–25 years) and age-matched uninfected controls observed a decrease in mature lymphocytes and CD34+ hematopoietic progenitors in the group with HIV, indicating an advanced aging profile [142,143]. Even more striking was the finding that the frequencies of immune components in young adults living with HIV mirrored the immune profiles of uninfected adults over 65 years of age and were associated with persistent viral replication [142]. These findings underscore the challenges of life-long adherence and its potential to accelerate immune aging in the population.

## 6. Conclusions

**Figure 2:**
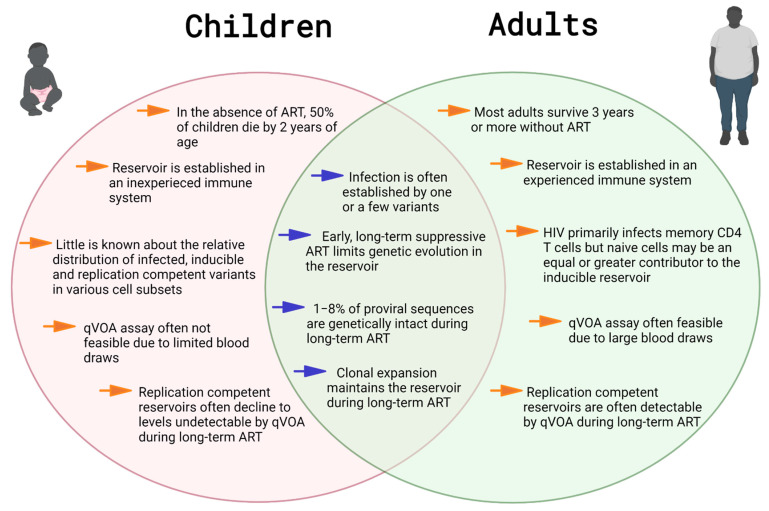
HIV-1 persistence in children vs. adults.

The immune system in early life provides a unique environment in which perinatal HIV infection is established. Although early treated children with viremia suppressed long-term on ART have smaller and less diverse HIV reservoirs, the proviral landscape during ART is reported to be similar to adults. The ability of early infected cells to persist long-term through clonal expansion poses a major barrier to cure. The effects of life-long HIV persistence and life-long ART are yet to be understood, but growing evidence suggests that these individuals are at an increased risk for developing non-AIDS-related co-morbidities, underscoring the need for an HIV cure (Figure 1 and Figure 2).
